# Persistent low avian malaria in a tropical species despite high community prevalence

**DOI:** 10.1016/j.ijppaw.2019.01.001

**Published:** 2019-01-11

**Authors:** Justin R. Eastwood, Lee Peacock, Michelle L. Hall, Michael Roast, Stephen A. Murphy, Anders Gonçalves da Silva, Anne Peters

**Affiliations:** a School of Biological Sciences, Monash University, 25 Rainforest Walk, Clayton, Victoria, 3800, Australia; b School of BioSciences, University of Melbourne, Melbourne, Parkville, Victoria, 3010, Australia; c Max Planck Institute for Ornithology, Vogelwarte Radolfzell, Schlossallee 2, D-78315, Radolfzell, Germany; d Adaptive NRM, Malanda, Queensland, 4885, Australia; e Research Institute for the Environment and Livelihoods, Charles Darwin University, Casuarina, Northern Territory, 0909, Australia; f Microbiological Diagnostic Unit Public Health Laboratory, Peter Doherty Institute for Infection and Immunity, The University of Melbourne, Australia

**Keywords:** Avian malaria, Wildlife diseases, Purple-crowned fairy-wrens, *Haemoproteus*, *Plasmodium*

## Abstract

Malarial and other haemosporidian parasites are widespread; however, their temporal dynamics are ill-understood. Longitudinal sampling of a threatened riparian bird revealed a consistently very low prevalence over 13 years (∼5%) despite infections persisting and prevalence increasing with age. In contrast, three key species within this tropical community were highly infected (∼20–75% prevalence) and these differences were stable. Although we found novel lineages and phylogenetic structure at the local level, there was little geographic structuring within Australasia. This study suggests that malarial parasite susceptibility is determined by host factors and that species can maintain low levels despite high community prevalence.

## Highlights

• Malarial parasite prevalence varied between species (∼5–75%).
• Persistent and low prevalence over 12y in a riparian bird.
• Oldest age category had highest parasite prevalence.
• Parasites showed genetic structure at the local level but not within Australasia.
• Tropical species can maintain low malarial parasite levels despite high exposure.

## Graphical abstract

**Figure:**
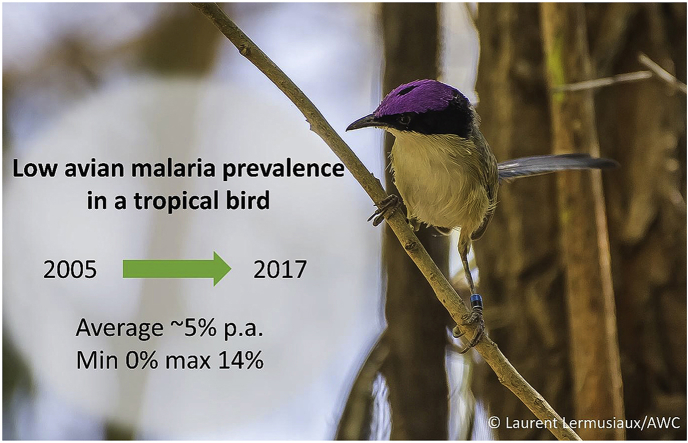

## 1 Introduction

The haemosporidian parasites that may cause the signs of malarial disease (Apicomplexa; Order Haemosporida; *Plasmodium*, *Haemoproteus* and *Leucocytozoon* spp.) can be highly prevalent in wildlife and have been implicated in several mass mortality events (van Riper et al., 1986; Valkiūnas, 2005). Because malarial parasites can reduce host condition (Merino et al., 2000; Marzal et al., 2008), reproduction and lifespan, they subsequently affect host fitness (Lachish et al., 2011b; Asghar et al., 2015). Hence, investigating the ecological relationships between hosts and parasites over time can yield insights into host-parasite co-evolution. While malarial parasite prevalence is known to be dynamic (e.g. Knowles et al., 2011), the consistency and drivers of such variation – in particular how prevalence varies over time within individuals, within populations and between host species in a community – are not well understood. This is despite the general acceptance that individual competence, population demographic factors, community interactions and temporal factors are all contributors in explaining malarial disease outbreaks (Valkiūnas, 2005). Furthermore, knowledge about phylogenetic diversity patterns of malaria parasites and prevalence are limited, because some regions are under-represented in the literature, notably the Australasian region, which is spatially and taxonomically isolated (Beadell et al., 2004; Clark et al., 2014).

The factors explaining individual variation in malarial parasite infection can often be attributed to individual state, like poor body condition, poor nutritional status or elevated stress levels (Crommenacker et al., 2011; Cornet et al., 2014), which can reduce host defences and increase the likelihood of being infected (Valkiūnas, 2005). In addition, infection may also be related to host genetics (Westerdahl et al., 2005; Bonneaud et al., 2006; Loiseau et al., 2011; Radwan et al., 2012) or demographic factors such as sex or age, e.g. older individuals tend to have a higher infection risk which is due to the increased chance of exposure over time or potentially immunosenescence (Wood et al., 2007). At the host species level, infection is also heterogeneous; for example, haemosporidian parasites have caused extinction (van Riper et al., 1986; Valkiūnas, 2005) and can be highly prevalent but seemingly benign in other host species (Bensch et al., 2007). In contrast, some species appear to have a very low prevalence, possibly related to a lack of vectors, life-history or population demographic factors (Kleindorfer et al., 2006; Balasubramaniam et al., 2013). Importantly, abiotic factors can explain a large proportion of the variation in prevalence both within and between species, because infection levels are often correlated with factors that affect the distribution and abundance of vectors (e.g. elevation, temperature, rainfall and water availability) and therefore parasite exposure (Wood et al., 2007; Laurance et al., 2013; Lalubin et al., 2013, Pulgarín-R et al., 2017). Host bird species vs. parasite co-evolutionary history (i.e. phylogeny), may influence prevalence as species may have different resistance or tolerance mechanisms against haemosporidians (Sorci, 2013). Alternatively, certain lineages may be more or less virulent between species (Schmid-Hempel, 2011). It is evident that many of the above factors involved in malarial parasite prevalence are temporally variable which suggests that prevalence should be dynamic. However, our knowledge about malarial parasite dynamics in wild animals is limited. Collecting longitudinal data within and between populations is advantageous because it can detect both transient and long-term parasite effects on populations and co-evolution.

We investigated malarial parasite dynamics over a 2–13 year period in four similarly sized (10–30 g) songbirds in the monsoonal tropics of northern Western Australia (Higgins and Peter, 2001, 2002). Three species [Purple-crowned fairy-wrens (*Malurus coronatus*, PCFW, *n* = 1387 samples; 815 individuals, 2005–2017), Buff-sided robins (*Poecilodryas cerviniventris,* BSR, *n* = 66 samples/individuals, 2007–2010) and White-gaped honey-eaters (*Lichenostomus unicolor*, WGH, *n* = 25 samples/individuals*,* 2007–2009)] are confined to the riparian habitat with dense vegetation and year-round free-standing water, enabling a permanent presence of potential vectors and a high force-of-infection (i.e. have a higher rate at which susceptible individuals are exposed and become infected). The fourth species [Red-backed fairy-wrens (*Malurus melanocephalus cruentatus,* RBFW, *n* = 78 samples/individuals, 2007–2008)] a congener of PCFW, inhabits the adjacent open savannah grasslands that lack water during the dry season and therefore experiences seasonal variation in the force-of-infection (Murphy et al., 2010). The focal species of this study, PCFW, are socially and genetically monogamous (Kingma et al., 2009; Hidalgo-Aranzamendi et al., 2016). They are cooperative breeders and nest year-round with distinct peaks in breeding activity associated with rainfall. Dominant PCFWs form stable territories with 1–6 subordinate individuals which are often retained offspring (Teunissen et al., 2018). We quantified (1) prevalence in four species, predicting high levels of infection in the three riparian specialists, in order to determine (2) whether individuals continue to harbour haemosporidian parasites after becoming infected and (3) evidence for within-species predictors of infection (age and sex) and temporal changes in infection (seasonal and annual). Finally, we investigated (4) lineage diversity at the local study site and at the Australasian region to determine whether there were any associations with host species or prevalence. Parasite lineage patterns associated with host species may indicate host-parasite co-adaptation or potential parasite-driven differences in species prevalence in an understudied region of the globe (Beadell et al., 2004; Clark et al., 2014). In addition, assessing lineage temporal stability or turnover rates over a long period of time is essential for understanding host species susceptibility and may provide insight into prevalence cycles (Fallon et al., 2004). Our aim was to determine the ecological predictors of the spread of infection over time and thus contribute to our understanding of host-parasite co-evolution.

## 2 Materials and methods

**Fig. 1:**
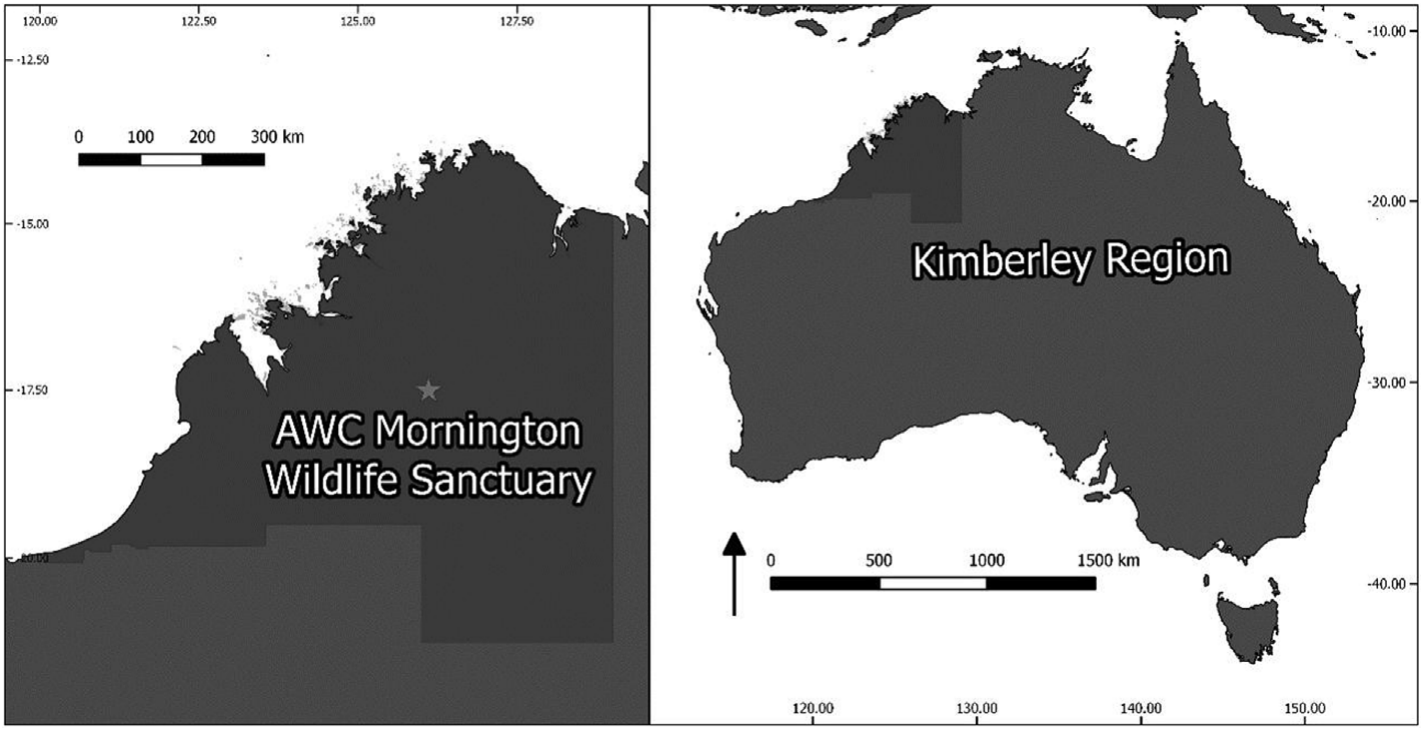
Map of Australia and the Kimberly region. Sampling was conducted at the Australian Wildlife Conservancy's Mornington Wildlife Sanctuary (17°31′S, 126°6’E). Star indicates the location of the field site where samples were collected.

Longitudinal sampling was conducted at the Australian Wildlife Conservancy's Mornington Wildlife Sanctuary (Fig. 1: 17°31′S, 126°6’E) along a 15 km stretch of the Adcock river and Annie Creek (Fan et al., 2017). As part of a long-term study investigating a broad range of ecological and evolutionary questions, this population has been monitored for 13 years. All birds were caught using mist nets, blood sampled [10–70 μl of blood stored in Longmire's lysis buffer (2005–2010) or ethanol (2011–2017)] and were marked with a metal identification ring (ABBBS) and unique colour bands (PCFW and RBFW). PCFWs are classified as endangered (Threatened Species Scientific Committee, 2015) and whilst the other species are not classified as threatened, the RBFW population has been reported to be declining (Murphy et al., 2010). PCFWs were sampled at multiple time periods over their lives [*n* = 105 nestlings 7 days old, free-flying birds ranging from age 1–139 months (1st year, *n* = 516; 2nd year, *n* = 134; 3rd year, *n* = 68; >4th year, *n* = 91)]. Sex was identified by plumage characteristics or molecular based techniques (Griffiths et al., 1998, Fan et al., 2018).

DNA was extracted from blood samples (Eastwood et al., 2018). To detect haemosporidian parasites we used a conventional PCR protocol which is described in the supplementary material. To assess the validity of the initial malaria detection PCR, we analysed blood smears from n = 138 PCFW individuals. To prepare smears, a small drop of blood was smeared onto a glass microscope slide and air dried in the field (Campbell, 2015). Once in the lab, the smear was immersed in 50% May-Grünwald and 10% Giemsa stain for 15 min each and then in distilled water for 5 min. The slides were then air dried and viewed under 1000 × magnification along the feathered edge as described in Campbell (2015). 22 samples were found to be positive using the malaria detection PCR, only one of which was found to be positive when screening blood smears. The other 116 samples were negative using both PCR and blood smears. The comparison suggests that there is agreement between the two methods (85% agreement, kappa ±SE = 0.074 ± 0.07) but strongly suggests that the PCR method is more sensitive at detecting malaria than blood smears (as previously shown, e.g. Waldenström et al., 2004). In addition to these tests, we thoroughly searched 4 blood smears with the knowledge they had tested positive using PCR. In all instances, they were found to also be positive in the smears with very low parasitaemia. Importantly, all positive blood smears were also positive in the PCR.

To prepare samples for sequencing, we used a nested PCR protocol which amplifies a 580bp region of the Cytochrome *b* gene and which amplifies *Haemoproteus*, *Plasmodium* and *Leucocytozoon*. Positive samples were then sent to Micromon (Monash University, Australia) for sequencing. All analyses conducted on the nucleotide sequence data were conducted using MEGA 7.0.26 (Kumar et al., 2016), trees were constructed using maximum likelihood approach. Sequences from the Australasian region were obtained from the MalAvi database (Bensch et al., 2009).

Statistical analyses were conducted using SPSS 23 (IBM). In brief, we compared species prevalence using a binary logistic regression model with infection status (presence/absence) as the dependent variable, year as a random effect with species and season (“wet” or “dry” denoting high and low rainfall periods respectively) included as fixed effects. From the PCFW dataset we selected a random sample from each individual that had been sampled multiple times to avoid pseudo-replication and excluded nestlings (n = 731 PCFW samples included out of n = 1387). Two WGH samples in 2010 and two RBFW samples in 2006 were excluded due to the low sample sizes. Age (in months), sex and year differences were tested using Chi square tests or Fisher's exact when expected values were less than five. See supplementary material for detailed materials and methods regarding the DNA extraction, PCR detection, statistical and phylogenetic analyses.

## 3 Results and discussion

**Fig. 2:**
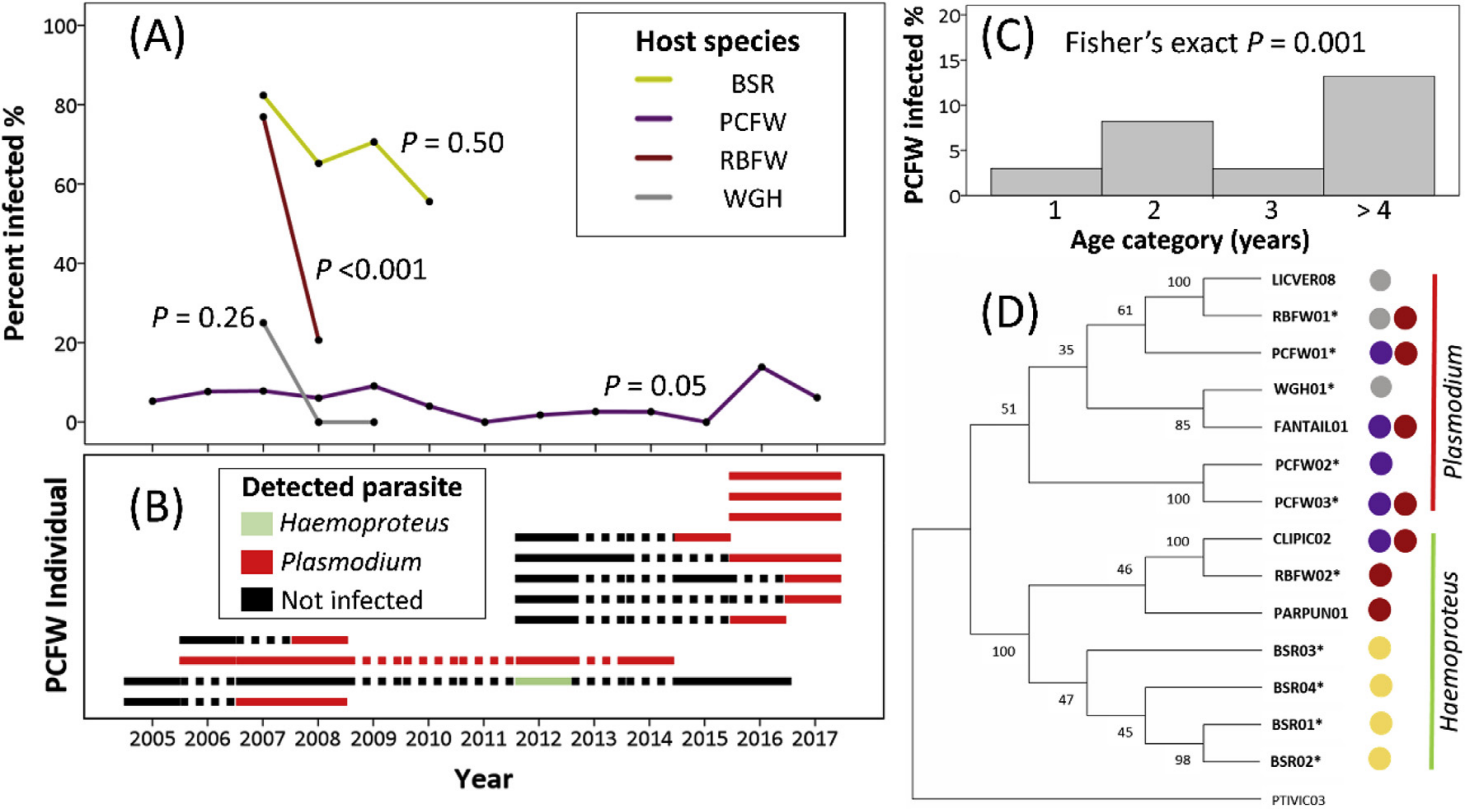
(A) Malarial parasite prevalence across years in four bird species, buff-sided robin (BSR, *n* = 66), purple-crowned fairy-wren (PCFW, *n* = 731), red-backed fairy-wren (RBFW, *n* = 78), white-gaped honeyeater (WGH, *n* = 25). Fisher's exact *P*-values test for annual differences in infection within each bird species. (B) Longitudinal sampling of infected PCFW adults (individuals presented were sampled more than twice and were identified as infected with *Haemoproteus* or *Plasmodium*). Dotted lines indicate uncertainty in years when no sample was available. Each individual was infected with a single lineage. (C) Percentage of individual PCFW infected within each age category. (D) Local phylogenetic relationship between parasite lineages, colours refer to host species as for (A). Maximum likelihood tree was inferred using GTR + G + I with 1000 bootstrap replicates; novel lineages are indicated by *. (For interpretation of the references to colour in this figure legend, the reader is referred to the Web version of this article.)

Overall haemosporidian prevalence varied significantly between bird species while controlling for year (Table S1) with BSR having the highest prevalence, followed by RBFW, WGH, and PCFW (Fig. 2A). The high prevalence in BSR consisted solely of *Haemoproteus*, while WGH were only infected by *Plasmodium* and fairy-wrens had both (Fig. S1). Bird species differences in overall infection rates were largely consistent across years (Fig. 2A) but were not qualitatively related to ecology or phylogeny: three species co-occurring within a narrow (10–30 m wide) riparian zone (BSR, WGH and PCFW) had highest and lowest prevalence, while the two congeneric fairy-wrens had lowest and second-highest prevalence. Although reduced sample sizes in all species except PCFW imply increased estimate error, particularly across years, the logistic regression and 95% confidence intervals show that the species effect is strong (Table S1). The differences between birds in prevalence imply that high force-of-infection resulting from proximity to year-round free-standing water with associated greater vector burden (Lachish et al., 2011a) cannot explain host species-level variability in prevalence in this system. Our findings, are in agreement with other studies which show that within the same geographic area hosts differ in prevalence (Fallon et al., 2003; Beadell et al., 2004, Pulgarín-R et al., 2017). Furthermore, we found no evidence to suggest that the annual wet and dry season influenced prevalence (Table S1), nor was there evidence for an interaction between host species and season. These results suggest that vector exposure is not the primary factor driving prevalence differences within or between host species.

Intra-specific patterns of prevalence were evident in PCFWs, where age affected likelihood of infection. No nestlings were infected which confirms findings in other passerine species (Cosgrove et al., 2006), although 5 out of 215 fledglings (1–2 months of age) were positive, showing that early infection is possible. Longitudinal sampling of individual PCFWs showed that individuals can also acquire infections any time in adult life (Fig. 2B; mean age = 44, range 5–117 months, n = 12) but do not subsequently lose the infection. Consequently, despite low prevalence overall (∼5%), increasing age was associated with greater infection, with individuals greater than four years, the oldest age class, having the highest prevalence (∼13%; Fig. 2C), which is consistent with other bird species (Wood et al., 2007; Knowles et al., 2011). Additionally, this pattern suggests that the consistently low prevalence in PCFW is not due to a prevailing ability to (rapidly) clear existing infections.

Low prevalence, when not explained by reduced vector exposure, may arise when co-adapted parasites are absent (Schmid-Hempel, 2011). Although the study population of PCFWs has doubled since 2010, the PCFWs previously underwent a decline in this population and across its range due to habitat degradation (Skroblin and Legge, 2012). Thus, we might speculate that fragmentation and isolation resulted in the loss of host-specific parasites in PCFWs, leaving this population exposed to less host-specific spill-over infections. This is consistent with the lack of unique and persistent *Haemoproteus* infections (one infected individual shared a lineage with RBFW Fig. 2B). However, although Plasmodiidae were not prevalent, individual infections were persistent long-term (up to >8.5 years; Fig. 2B). Unlike *Haemoproteus*, *Plasmodium* infections were not cleared, and individuals remained infected with a single lineage, which may be evidence for parasite co-adaptation. This may be evidence to support long term chronic infections of *Plasmodium* in PCFWs, however information on parasitaemia is needed to properly determine disease stage (Asghar et al., 2012). The *Haemoproteus* infection may have been incidental. Therefore, the overall low and consistent prevalence in PCFW is also not easily explained by loss of adapted parasites, and other explanations, e.g. first-line immune defences, vector avoidance strategies, or bird species preference by vectors need to be considered (Cornet et al., 2013; De Moraes et al., 2014; Clark et al., 2016).

**Fig. 3:**
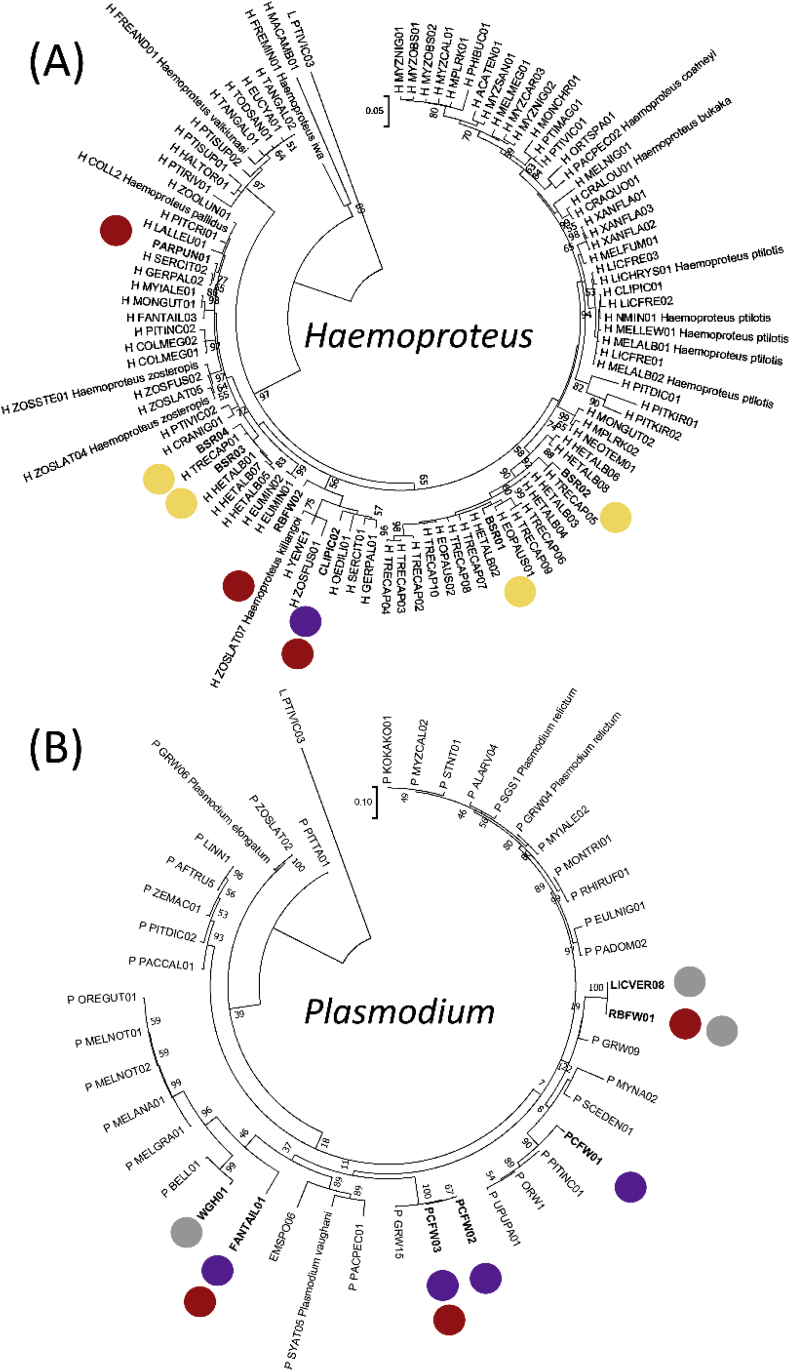
Maximum likelihood phylogenetic inference of (A) *Haemoproteus* and (B) *Plasmodium* from the Australasian region. Sequences were included if they were at least 479 nucleotides in length and were found to be unique from a pairwise distance analysis (see methods). Bootstrap support values are shown if greater than 50. Dots indicate the 14 lineages that were detected in this study and their colour denotes the bird species they occurred within [Purple = PCFW (*M. c. coronatus*), Red = RBFW (*M. melanocephalus*), Yellow = BSR (*P. cerviniventris*), Grey = WGH (*L. unicolor*)]. (For interpretation of the references to colour in this figure legend, the reader is referred to the Web version of this article.)

In addition to host factors, parasite lineages may have a role in determining prevalence, although within this parasite-rich community the phylogenetic patterns were complex. We identified 14 parasite lineages, 7 each of *Plasmodium* and *Haemoproteus* spp., 10 of which were novel (Fig. 2D). All individuals were infected with a single lineage throughout infection (Fig. 2B). Local phylogenetic patterns (Fig. 2D) were evident within the *Haemoproteus* clade, with two main groups: one consisting solely of lineages from BSR and the other shared between the two fairy-wrens (PCFW and RBFW). There was little support for structure within the *Plasmodium* clade as branch support was low and lineages were shared between host species. While we cannot completely rule out lineage diversity accounting for prevalence variability between birds, it is unlikely because lineages infect multiple hosts, particularly in *Plasmodium* spp. (e.g. between RBFW, PCFW and WGH; Fig. 2D). Parasite nucleotide diversity and lineage diversity varied between host species (Fig. 3A and B): WGH (mean p-distance d = 0.060, Simpson's index I = 1.00) had the highest followed by RBFW (d = 0.047, I = 0.73) and PCFW (d = 0.041, I = 0.59). BSR had the lowest diversity (d = 0.028, I = 0.59) and the highest prevalence (Fig. 2A) which may reflect a recent outbreak or tight host-parasite co-adaption. Compared with malaria lineages across Australasia, there is no evidence that lineages from our study site are spatially unique or host specific (*Haemoproteus,* Fig. 3A; *Plasmodium,* Fig. 3B), indicating host species generalists and/or frequent host spill-over (Ricklefs et al., 2004; Ewen et al., 2012). In addition, parasite lineages from PCFW, RBFW and WGH were found previously in different bird species and across a large geographic area (Table S2). More regional structure was present in the *Haemoproteus* phylogeny as previously observed (e.g. Beadell et al., 2004). Why patterns emerge at the local but not regional levels may be a consequence of not accounting for co-adaptation to vectors, detecting spill-over into non-competent hosts rather than co-adapted lineages or evolutionary associations across time and space, all of which would introduce error. While this study adds to the overall phylogenetic picture of avian malaria phylogeography, more sampling in under-represented regions is needed, notably the Australasian region in order to understand the high level of diversity observed (Beadell et al., 2004; Clark et al., 2014).

This study found extreme and consistent variation in avian malaria prevalence between sympatric, ecologically similar, birds, however the explanation as to why this is the case is complex. In this tropical community, we assume that vector abundance is typically high year-round, due to the year-round presence of water, and should correlate with malarial parasite exposure. We expected that bird species within the riparian zone would have similar and higher prevalence compared to the grassland bird species but instead found a high degree of variability, with both the least and highest infected occurring within the riparian zone. Furthermore, we expected to observe that PCFWs, because they inhabit a wetter environment (associated vector exposure), would have a higher prevalence compared to RBFWs, despite their close phylogenetic relationship. However, we found that the opposite was true with a higher prevalence in RBFWs. Notably, habitat (with free-standing water or mostly dry) and season (wet or dry) seemed to have little impact on prevalence, contrary to what other studies have shown (Cosgrove et al., 2008; Hernández-Lara et al., 2017), and may suggest that vector exposure is consistent (Valkiūnas, 2005). The observed prevalence differences between bird species are mostly consistent and it is unlikely that parasite lineages explain this prevalence variability because parasite lineages can infect multiple birds. Overall, our results suggest that while within-species variation is determined by ecological (e.g. annual differences) or individual (e.g. age) factors, between-species prevalence is determined not by ecology but by vector preferences (Pulgarín-R et al., 2017) or by unknown host factors such as behaviour (Beadell et al., 2004, Pulgarín-R et al., 2017). PCFWs are long-lived; occur at high density; live within a year-round parasite-rich community with year-round presence of vectors; are susceptible to a diversity of lineages and can maintain infections for very long periods. Why then PCFWs have such a low prevalence, despite combining so many attributes conducive to infection, is an interesting puzzle for future studies.

## References

[bib1] Asghar M. (2012). Primary peak and chronic malaria infection levels are correlated in experimentally infected great reed warblers. Parasitology.

[bib2] Asghar M. (2015). Hidden costs of infection: chronic malaria accelerates telomere degradation and senescence in wild birds. Science.

[bib3] Balasubramaniam S. (2013). Prevalence and diversity of avian haematozoa in three species of Australian passerine. Emu.

[bib4] Beadell J.S. (2004). Prevalence and differential host-specificity of two avian blood parasite genera in the Australo-Papuan region. Mol. Ecol..

[bib5] Bensch S. (2007). Temporal dynamics and diversity of avian malaria parasites in a single host species. J. Anim. Ecol..

[bib6] Bensch S. (2009). MalAvi: a public database of malaria parasites and related haemosporidians in avian hosts based on mitochondrial cytochrome b lineages. Mol Ecol Resour.

[bib7] Bonneaud C. (2006). Major histocompatibility alleles associated with local resistance to malaria in a passerine. Evolution.

[bib8] Campbell T.,W. (2015). Exotic Animal Hematology and Cytology.

[bib9] Clark N.J. (2014). A review of global diversity in avian haemosporidians (*Plasmodium* and *Haemoproteus*: Haemosporida): new insights from molecular data. Int. J. Parasitol..

[bib10] Clark N.J. (2016). Migration strategy and pathogen risk: non-breeding distribution drives malaria prevalence in migratory waders. Oikos.

[bib11] Cornet S. (2013). Malaria infection increases bird attractiveness to uninfected mosquitoes. Ecol. Lett..

[bib12] Cornet S. (2014). Impact of host nutritional status on infection dynamics and parasite virulence in a bird-malaria system. J. Anim. Ecol..

[bib13] Cosgrove C.L. (2006). No evidence for avian malaria infection during the nestling phase in a passerine bird. J. Parasitol..

[bib14] Cosgrove C.L. (2008). Seasonal variation in Plasmodium prevalence in a population of blue tits Cyanistes caeruleus. J. Anim. Ecol..

[bib15] van de Crommenacker J. (2011). Parasitic infection and oxidative status are associated and vary with breeding activity in the Seychelles warbler. Proc. R. Soc. B.

[bib16] De Moraes C.M. (2014). Malaria-induced changes in host odors enhance mosquito attraction. Proc. Natl. Acad. Sci. Unit. States Am..

[bib17] Eastwood J.R. (2018). Increasing the accuracy and precision of relative telomere length estimates by RT qPCR. Mol Ecol Resour.

[bib18] Ewen J.G. (2012). Establishment of exotic parasites: the origins and characteristics of an avian malaria community in an isolated island avifauna. Ecol. Lett..

[bib19] Fallon S.M. (2003). Island and taxon effects in parasitism revisited: avian malaria in the lesser antilles. Evolution.

[bib20] Fallon S.M. (2004). Temporal stability of insular avian malarial parasite communities. Proc. R. Soc. Lond. B Biol. Sci..

[bib21] Fan M. (2017). No fitness benefits of early molt in a fairy-wren: relaxed sexual selection under genetic monogamy?. Behav. Ecol..

[bib22] Fan M. (2018). From ornament to armament or loss of function? Breeding plumage acquisition in a genetically monogamous bird. J. Anim. Ecol..

[bib23] Griffiths R. (1998). A DNA test to sex most birds. Mol. Ecol..

[bib24] Hernández-Lara C. (2017). Spatial and seasonal variation of avian malaria infections in five different land use types within a Neotropical montane forest matrix. Landsc. Urban Plann..

[bib25] Hidalgo-Aranzamendi N. (2016). Incest avoidance, extrapair paternity, and territory quality drive divorce in a year-round territorial bird. Behav. Ecol..

[bib26] Higgins P.J., Peter J.M. (2001). Handbook of Australian, New Zealand and Antarctic Birds. Volume 5: Tyrant-Flycatchers to Chats.

[bib27] Higgins P.J., Peter J.M. (2002). Handbook of Australian, New Zealand and Antarctic Birds. Volume 6: Pardalotes to Shrike- Thrushes.

[bib28] Kingma S.A. (2009). Radical loss of an extreme extra-pair mating system. BMC Ecol..

[bib29] Kleindorfer S. (2006). Ticks (*Ixodes* sp.) and blood parasites (*Haemoproteus* spp.) in New Holland Honeyeaters (*Phylidonyris novaehollandiae*): evidence for site specificity and fitness costs. Emu.

[bib30] Knowles S.C.L. (2011). Molecular epidemiology of malaria prevalence and parasitaemia in a wild bird population. Mol. Ecol..

[bib31] Kumar S. (2016). MEGA7: molecular evolutionary genetics analysis version 7.0 for bigger datasets. Mol. Biol. Evol..

[bib32] Lachish S. (2011). Infection dynamics of endemic malaria in a wild bird population: parasite species-dependent drivers of spatial and temporal variation in transmission rates. J. Anim. Ecol..

[bib33] Lachish S. (2011). Fitness effects of endemic malaria infections in a wild bird population: the importance of ecological structure. J. Anim. Ecol..

[bib34] Lalubin F. (2013). Temporal changes in mosquito abundance (*Culex pipiens*), avian malaria prevalence and lineage composition. Parasites Vectors.

[bib35] Laurance S.G.W. (2013). Habitat fragmentation and ecological traits influence the prevalence of avian blood parasites in a tropical rainforest landscape. PLoS One.

[bib36] Loiseau C. (2011). Plasmodium relictum infection and MHC diversity in the house sparrow (*Passer domesticus*). Proc. R. Soc. Lond. B Biol. Sci..

[bib37] Marzal A. (2008). Effects of malaria double infection in birds: one plus one is not two. J. Evol. Biol..

[bib38] Merino S. (2000). Are avian blood parasites pathogenic in the wild? A medication experiment in blue tits (*Parus caeruleus*). Proc. R. Soc. Lond. B Biol. Sci..

[bib39] Murphy S.A. (2010). The effects of early and late-season fires on mortality, dispersal, physiology and breeding of red-backed fairy-wrens (*Malurus melanocephalus*). Wildl. Res..

[bib40] Pulgarín‐R P.C. (2017). Host species, and not environment, predicts variation in blood parasite prevalence, distribution, and diversity along a humidity gradient in northern South America. Ecol Evol.

[bib41] Radwan J. (2012). MHC diversity, malaria and lifetime reproductive success in collared flycatchers. Mol. Ecol..

[bib42] Ricklefs R.E. (2004). Evolutionary relationships, cospeciation, and host switching in avian malaria parasites. Syst. Biol..

[bib43] Schmid-Hempel P. (2011). Evolutionary Parasitology: the Integrated Study of Infections, Immunology, Ecology, and Genetics.

[bib44] Skroblin A., Legge S. (2012). Influence of fine-scale habitat requirements and riparian degradation on the distribution of the purple-crowned fairy-wren (*Malurus coronatus coronatus*) in northern Australia. Austral Ecol..

[bib45] Sorci G. (2013). Immunity, resistance and tolerance in bird–parasite interactions. Parasite Immunol..

[bib46] Teunissen N. (2018). More than kin: subordinates foster strong bonds with relatives and potential mates in a social bird. Behav. Ecol..

[bib47] Threatened Species Scientific Committee (2015). Conservation Advice *Malurus coronatus Coronatus* (Purple-crowned Fairy-Wren (Western)).

[bib48] Valkiūnas G. (2005). Avian Malaria Parasites and Other Haemosporidia.

[bib49] van Riper C. (1986). The epizootiology and ecological significance of malaria in Hawaiian land birds. Ecol. Monogr..

[bib50] Waldenström J. (2004). A new nested polymerase chain reaction method very efficient in detecting Plasmodium and Haemoproteus infections from avian blood. J. Parasitol..

[bib51] Westerdahl H. (2005). Associations between malaria and MHC genes in a migratory songbird. Proc. R. Soc. Lond. B Biol. Sci..

[bib52] Wood M.J. (2007). Within-population variation in prevalence and lineage distribution of avian malaria in blue tits, *Cyanistes caeruleus*. Mol. Ecol..

