# Supplementary material for: Persistent low avian malaria in a tropical species despite high community prevalence
Source: Int J Parasitol Parasites Wildl. 2019 Jan 11;8:88–93. doi: 10.1016/j.ijppaw.2019.01.001 (PMC6350384; doi:10.1016/j.ijppaw.2019.01.001)

# Supplementary materials and methods

## Malarial parasite detection and barcoding

DNA was extracted from a small dried flake of blood using the QIAgen DNeasy kit protocol except for the following minor volume adjustments for samples collected between 2005-2006 and 2011-2014: blood was added into 25μl of Proteinase K and 200μL of Buffer AL, incubation was at 56^O^C for 4 hours then overnight at 37 ^O^C and 200μL of Phosphate Buffered Solution (pH7.2). The elution was completed using 80μl of Buffer AE. Samples from 2007-2010 and 2015-2017 were similarly extracted using QIAcube automated DNA extraction method as per Eastwood et al. 2018. DNA quantification and quality were assessed using a Nanodrop ND-1000.

To determine prevalence, we used a general Haematozoon PCR detection method which was specifically designed for use in the Australasian region. Using the program PriFi (Fredslund et al. 2005), primers were designed by alignment of closed mitogenomes (*Leucocytozoon caulleryi* — NC_015304.1; *Plasmodium berghei* — NC_015303.1; *Haemoproteus columbae* — NC_012448.1; and *Parahaemoproteus vireonis* — NC_012447.1). The top primer pairs were tested using a 10-fold serial dilution of a 1 ng/µl *P. falciparum* 3D7 DNA sample (obtained from the Department of Microbiology at Monash University). The primers chosen for this study (MalMitoF1 5’-AGCCAAAAGAATAGAAACAGATGCCAGGCCAA–3’ and MalMitoR1 5’-AGCGATRCGTGAGCTGGGTTAAGAACGTCTTGAG–3’) proved to be highly sensitive and detected as few as 12 copies of *P. falciparum* 3D7 DNA. In the *P. berghei* genome, MalMitoR1 and MalMitoF1 sit in LS3 (Forward) and LS9 (Reverse) rRNA large subunits, and the amplicon spans the 3' region of LS3, the intra-gene region between LS3 and LS9, and the 5' region of LS9, capturing positions 1443 to 1558 of the mitochondrial genome. Each reaction volume was 10µl containing 5µl of HotStart Master Mix (Qiagen), 0.05 µM of each primer (MalMitoF1 and MalMitoR1) and 30ng of DNA. PCR conditions were as follows: 95 ^O^C for 15 min, 5 cycles [94 ^O^C for 30 sec, 65 ^O^C for 30 sec, 72 ^O^C for 30 sec], 10 cycles [94 ^O^C for 30 sec, 65* ^O^C for 30 sec, 72 ^O^C for 30 sec] and 30 cycles [94 ^O^C for 30 sec, 55 ^O^C for 30 sec, 72 ^O^C for 30 sec]. The symbol (*) denotes a 1°C drop in annealing temperature with each cycle. PCR products were run on a 2% agarose gel at 90V for 45 minutes. DNA extraction method (95.45% agreement, Kappa ± SE = 0.90 ± 0.098, *n* = 22) and blood storage buffer (100% agreement, Kappa = 1.00, *n* = 14) had no effect on malaria detection. Positive samples were then barcoded for lineage identification using a nested PCR method and sequencing of the final products (except PCFW samples for which we only used the second PCR of the nested protocol). The first reaction of the nested PCR amplifies a 580bp product of the Cytochrome b gene using the primer combination Prim3_F2 5’–ACTGGTGTATTATTAGCAACTTGTTATACT–3’ and Prim3_R1 5’–GCTTGGGAGCTGTAATCATAATGT–3’. The Prim3 primer set was designed to improve the amplification of the Haem primers (below) by flanking the barcoding region identified in Waldenström et al. (2004). Prim3 primers were designed by first aligning 10 Haemosporidia *cytb* sequences using Clustal Omega (1.2.0) and creating a consensus sequence using DAMBE (v5.0). The primers were then generated using the online tool Primer3 (Untergasser et al. 2012). Total reaction volume was 25µl which contained 12.5µl of HotStart Master Mix, 0.75 mM MgCl_2_, 2.5 µM primer concentration and 30ng of DNA. PCR conditions were as follows: 95 ^O^C for 15 min, 15 cycles [94 ^O^C for 30 sec, 65* ^O^C for 60 sec, 70 ^O^C for 70 sec] and 30 cycles [94 ^O^C for 30 sec, 45 ^O^C for 60 sec, 70 ^O^C for 70 sec]. Products were run on a 1 % agarose gel to check amplification success and determine product dilutions in the nested PCR. Samples with zero or faint bands had 5ul of template DNA diluted in 45µl of milliQ water. Samples with clear bands had 5µl of PCR product diluted in 95µl of milliQ water. The second PCR reaction contained 3ul of the diluted PCR products, 12.5µl of HotStart Master Mix, 0.75 mM MgCl_2_, 2.5 µM primer concentration in a total reaction volume of 25µl. The second primer combination was HaemNF 5’–CATATATTAAGAGAATTATGGAG–3’ and HaemNR2 5’–AGAGGTGTAGCATATCTATCTAC–3’ and was used for sequencing to determine the specific parasite lineage (Waldenström et al. 2004). PCR conditions for the second reaction were: 95 ^O^C for 15 min, 5 cycles [94 ^O^C for 30 sec, 55* ^O^C for 45 sec, 70 ^O^C for 70 sec], 35 cycles [94 ^O^C for 30 sec, 50 ^O^C for 45 sec, 70 ^O^C for 70 sec] and 70 ^O^C for 10 min. PCR products were run on a 1% agarose gel. Positive samples (*n* = 101 out of 146) were diluted and sent for sequencing at Micromon (Monash University, Australia). A nucleotide blast analysis suggests that the primer sequences used in this study are highly specific to *Plasmodium*, *Haemoproteus* and *Leucocytozoon* as evidenced by the high sequence homology of the primers (Apicomplexans: 97-100% identity across >50% of the region). In addition, the primers are suitable for detecting a range of avian haemosporidians in the Australasian region as they have been used in over 60 wild bird species and have successfully amplified and sequenced 169 lineages corresponding to either *Haemoproteus*, *Plasmodium* or *Leucocytozoon* (Peacock, L., Gonçalves da Silva, A., Clarke, RH., unpublished data). Some positive samples found using the detection PCR failed to amplify using the nested barcoding primers (*N* = 39 samples from PCFW, *n* = 5 RBFW and *n* = 1 WGH). This may indicate that the initial detection PCR is more sensitive than the barcoding PCRs. Alternatively, it is possible that the detection primers are amplifying protozoa other than *Plasmodium*, *Haemoproteus* or *Leucocytozoon* (e.g. from the genera *Hepatocystis*, *Nycteria*, *Cyclospora* or *Eimeria*).

## Phylogenetic analyses

Sequencing reads from the forward and reverse primers were aligned using Geneious (Version 11.1.4, Biomatters) and the consensus sequence uploaded onto the MalAvi (Bensch et al. 2009) and GenBank databases (See supplementary material). Avian malaria sequences from the Australasian region were found by searching MalAvi and Genbank databases and exported to MEGA version 7.0.26 (Kumar et al. 2016) for alignment with sequences produced in this study and further analysis. The Muscle function with default settings was used to align the sequences. For analysis, sequences were trimmed to 479 nucleotide region and those that had gaps were removed from analysis. To identify novel lineages, we conducted a nucleotide BLAST search on the NCBI database and conducted a pairwise distance analysis. Non-unique lineages were excluded from further analyses. We conducted all phylogenetic analyses using only unique lineages. First, we assessed the phylogenetic relationship between lineages that were sampled at our field site using a Maximum likelihood approach with a General Time Reversible model (GTR) + Gamma distributed rate variation across sites (Γ) + invariant sites (I; chosen based on the AIC model selection function (default settings) within MEGA) and 1000 bootstrap replicates. Second, we determined the phylogenetic relationship between our local sequences and those from MalAvi found in the Australasian region separately for *Haemoproteus* and *Plasmodium* using a Maximum likelihood approach with 1000 bootstrap replicates, a GTR + Γ + I and Tamura-Nei + Γ + I models, respectively. All trees were rooted using an archived *Leucocytozoon* sequence from the MalAvi database L_PTIVIC03. Lineage diversity was analysed for each bird species in two distinct ways. First, we estimated p-distance (the proportion of nucleotide sites that are distinct) using the nucleotide distance function in MEGA. Second, we calculated Simpson’s diversity index of the lineages within each bird species.

## Statistical analyses

All statistical analyses were conducted using SPSS version 23. To investigate whether malarial parasite prevalence differed between the four bird species we modelled overall infection status (present/absent) using a binary generalized linear mixed effects model with a logit link function. Year was included as a random effect with bird species and season (“wet” or “dry” denoting times of year with high and low rainfall respectively) included as fixed effects. We included an interaction between bird species and season, but this was non-significant and subsequently removed from the analysis. For PCFWs we randomly selected a single sample if individuals were screened multiple times (*n* = 815). Nestlings were removed from the analyses as they were all negative and it is biologically unlikely that malaria detection is possible at 7 days old (PCFW *n* = 731). For testing prevalence across years, we excluded *n* = 2 WGH samples in 2010 and *n* = 2 RBFW samples in 2006 due to the extremely low sample sizes. In all other birds individuals were only sampled once. To investigate the predictors of malaria infection in PCFWs we ran a logistic regression with individual as a random effect to account for longitudinal sampling of individuals and included the fixed terms year, sex and age category (1^st^ year, 2^nd^ year, 3^rd^ year and > 4^th^ year). However, individual explained zero variance and including year caused quasi-complete separation within the data because there were too few positive samples. Therefore, we randomly selected a single sample from each individual that was sampled more than once as above and used simpler analyses as described below. Within each host species we tested whether prevalence varied between years (all bird species), sex (PCFW and RBFW) and age (PCFW) using Chi square tests. Sex and age data were not available for BSR or WGH so only year was tested for an association with infection status whilst age sampled was not known for RBFW. When expected values were less than five, we used Fisher’s exact test for association instead.

# Supplementary References

Bensch, S. et al. 2009. MalAvi: a public database of malaria parasites and related haemosporidians in avian hosts based on mitochondrial cytochrome b lineages. - Mol Ecol Resour 9: 1353–1358.

Eastwood, J. R. et al. 2018. Increasing the accuracy and precision of relative telomere length estimates by RT qPCR. - Mol Ecol Resour 18: 68–78.

Kumar, S. et al. 2016. MEGA7: Molecular Evolutionary Genetics Analysis version 7.0 for bigger datasets. - Mol Biol Evol 33: 1870–1874.

Untergasser, A. et al. 2012. Primer3—new capabilities and interfaces. - Nucleic Acids Research 40: e115–e115.

Waldenström, J. et al. 2004. A new nested polymerase chain reaction method very efficient in detecting Plasmodium and Haemoproteus infections from avian blood. - J Parasitol 90: 191–194.

# Supplementary results

**Table S1**: Host species was a significant predictor of haemosporidian prevalence whilst controlling for year sampled as a random effect and season (“wet” or “dry” denoting high and low rainfall periods respectively).

| **Fixed effect** | **Coefficient** | **Standard error** | **95% confidence intervals** | **t** | ***P*** |
| --- | --- | --- | --- | --- | --- |
| Intercept | -2.27 | 0.72 |  | -3.17 | 0.002 |
| Species |  |  |  |  |  |
| BSR (*P. cerviniventris*) | 3.17 | 0.71 | 1.784 – 4.57 | 4.48 | <0.001 |
| PCFW (*M. c. coronatus*) | -0.55 | 0.69 | -1.90 –0.81 | -0.80 | 0.43 |
| RBFW (*M. melanocephalus*) | 1.64 | 0.71 | 0.24 – 3.03 | 2.30 | 0.022 |
| WGH (*L. unicolor*) | 0 | - | - | - | - |
| Season (dry) | -0.33 | 0.29 | -0.91 – 0.24 | -1.14 | 0.26 |
| Year (random) | 0.49 | 0.32 |  | 1.54 | 0.12 |

**Table S2:** Four passerine species in northwest Australia [PCFW (*M. c. coronatus*), RBFW (*M. melanocephalus*), BSR (*P. cerviniventris*), WGH (*L. unicolor*); Mornington Wildlife Sanctuary] hosted 14 lineages of parasites. N represents the number of samples that were detected in this study. Four lineages have previously been discovered (100% nucleotide match using BLAST search) and those MalAvi ID (Bensch et al. 2009), locations and bird species are identified in bold font. WA = Western Australia, VIC = Victoria, QLD = Queensland.

| **Lineage number** | **N** | **Haemosporidian genus** | **Genbank Accession** | **MalAvi ID** | **Bird species** | **Location** |
| --- | --- | --- | --- | --- | --- | --- |
| 1 | 8 | *Haemoproteus* | MK374286 | BSR01 | *P. cerviniventris* | WA |
| 2 | 27 | *Haemoproteus* | MK374289 | BSR02 | *P. cerviniventris* | WA |
| 3 | 10 | *Haemoproteus* | MK374288 | BSR03 | *P. cerviniventris* | WA |
| 4 | 1 | *Haemoproteus* | MK374287 | BSR04 | *P. cerviniventris* | WA |
| 5 | 1 | *Haemoproteus* | MK374285 | RBFW02 | *M. melanocephalus* | WA |
| 6 | 8 | *Haemoproteus* | KC807994 | **CLIPIC02** | *M. c. coronatus*, *M. melanocephalus*, ***Climacteris picumnus*** | WA, **VIC** |
| 7 | 9 | *Haemoproteus* | KC807993 | **PARPUN01** | *M. melanocephalus*, ***Climacteris picumnus, Pardalotus punctatus*** | WA, **VIC** |
| 8 | 4 | *Plasmodium* | MK374281 | PCFW01 | *M. c. coronatus* | WA |
| 9 | 20 | *Plasmodium* | MK374282 | PCFW02 | *M. c. coronatus* | WA |
| 10 | 5 | *Plasmodium* | MK374283 | PCFW03 | *M. c. coronatus*, *M. melanocephalus* | WA |
| 11 | 2 | *Plasmodium* | MK374284 | RBFW01 | *M. melanocephalus*, *L. unicolor* | WA |
| 12 | 4 | *Plasmodium* | AY714196 | **FANTAIL01** | *M. c. coronatus*, *M. melanocephalus*, ***Rhipidura rufifrons***, ***Heteromyias albispecularis***, ***Pachycephala simplex***, ***Sericornis magnirostra***, ***Acridotheres tristis, Calidris tenuirostris*** | WA, **QLD** |
| 13 | 1 | *Plasmodium* | JQ905578 | **LICVER08** | *L. unicolor*, ***Lichenostomus*** ***versicolor***, ***Conopophila albogularis***, ***Myiagra Alecto***, ***Myzomela erythrocephala***, ***Myzomela obscura***, ***Passer domesticus***, ***Rhipidura leucophrys*** | WA |
| 14 | 1 | *Plasmodium* | MK374280 | WGH01 | *L. unicolor* | WA |

**Figure S1**: Lineage prevalence within each of the four bird species sampled. Blocks containing dots denote that the lineage belongs to the genus *Plasmodium*, whilst solid denotes *Haemoproteus*. Lineage numbers correspond to those in Table S2.


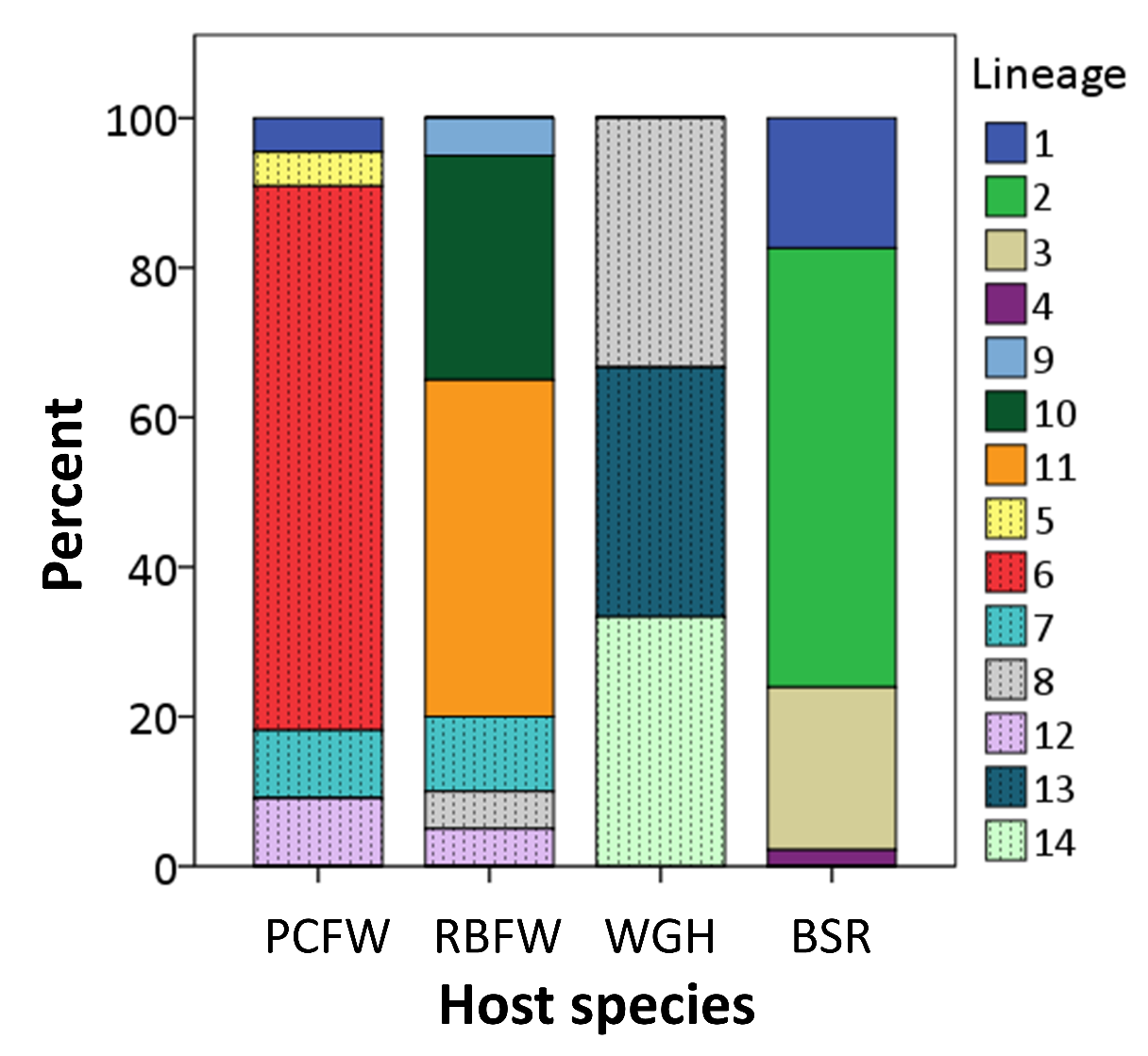

Supplement: Multimedia component 1 [file mmc1.docx]
